# Forkhead-Box-P3 Gene Transfer in Human CD4^+^ T Conventional Cells for the Generation of Stable and Efficient Regulatory T Cells, Suitable for Immune Modulatory Therapy

**DOI:** 10.3389/fimmu.2017.01282

**Published:** 2017-10-12

**Authors:** Laura Passerini, Rosa Bacchetta

**Affiliations:** ^1^Mechanisms of Peripheral Tolerance Unit, San Raffaele Telethon Institute for Gene Therapy (SR-TIGET), IRCCS San Raffaele Scientific Institute, Milan, Italy; ^2^Department of Stem Cell Transplantation and Regenerative Medicine, Division of Pediatrics, Stanford School of Medicine, Stanford, CA, United States

**Keywords:** regulatory T cells, forkhead box P3, tolerance, regulatory T cell-based cell therapy, gene transfer, antigen specificity, autoimmunity, immune dysregulation

## Abstract

The development of novel approaches to control immune responses to self- and allogenic tissues/organs represents an ambitious goal for the management of autoimmune diseases and in transplantation. Regulatory T cells (Tregs) are recognized as key players in the maintenance of peripheral tolerance in physiological and pathological conditions, and Treg-based cell therapies to restore tolerance in T cell-mediated disorders have been designed. However, several hurdles, including insufficient number of Tregs, their stability, and their antigen specificity, have challenged Tregs clinical applicability. In the past decade, the ability to engineer T cells has proven a powerful tool to redirect specificity and function of different cell types for specific therapeutic purposes. By using lentivirus-mediated gene transfer of the thymic-derived Treg transcription factor forkhead-box-P3 (FOXP3) in conventional CD4^+^ T cells, we converted effector T cells into Treg-like cells, endowed with potent *in vitro* and *in vivo* suppressive activity. The resulting CD4^FOXP3^ T-cell population displays stable phenotype and suppressive function. We showed that this strategy restores Treg function in T lymphocytes from patients carrying mutations in *FOXP3* [immune-dysregulation, polyendocrinopathy, enteropathy, X-linked (IPEX)], in whom CD4^FOXP3^ T cell could be used as therapeutics to control autoimmunity. Here, we will discuss the potential advantages of using CD4^FOXP3^ T cells for *in vivo* application in inflammatory diseases, where tissue inflammation may undermine the function of natural Tregs. These findings pave the way for the use of engineered Tregs not only in IPEX syndrome but also in autoimmune disorders of different origin and in the context of stem cell and organ transplantation.

## Introduction

Regulatory T cells (Tregs) are a subset of T lymphocytes devoted to the modulation of immune responses and to the maintenance of immunological tolerance. They control aberrant immune responses toward a wide range of antigens (Ags), including self-, food-Ags, allergens, and tumors (1). Several subsets of Tregs have been identified. Among those, Tregs expressing the forkhead-box-P3 (FOXP3) transcription factor (FOXP3^+^-Tregs) (2, 3) and the IL-10-dependent T-regulatory-type-1 cells (4) are the best characterized. The latter will be the subject of a review by Gregori et al. in the present Research Topic, whereas the former subset and its application in the clinical practice will be discussed here.

FOXP3^+^-Tregs can originate either in the thymus [thymic-derived Tregs (tTregs)] or differentiate in the periphery from naïve T cells (pTregs) (5, 6). Regardless of their origin, both subsets are characterized by constitutive expression of FOXP3, a transcription factor critical for their function, as demonstrated by the devastating autoimmunity resulting from mutations of *FOXP3* (7, 8). Impaired Treg function is the key pathogenic event leading to disruption of self-tolerance in patients with immune-dysregulation, polyendocrinopathy, enteropathy, X-linked (IPEX) syndrome (9, 10).

It is now well accepted that although FOXP3 expression is dispensable for thymic development of tTregs, mainly dictated by epigenetic remodeling occurring regardless of FOXP3, its expression becomes fundamental in later stages for the peripheral function and maintenance of Tregs (11). Indeed, high and stable FOXP3 expression allows the acquisition of full suppressive function and stability of the Treg lineage by orchestrating the expression or repression of multiple genes indispensable for Treg suppressive function (12–14).

In addition to FOXP3, the expression of several molecules, including high CD25 (IL2Rα chain) in the absence of CD127 (IL7Rα chain) (15), CTLA-4 (16), GITR (17), CD39 (18), Galectin 10 (19), latency-associated peptide (20), Helios (21), the T-cell immune receptor TIGIT (22), and glycoprotein-A repetitions predominant (23) has been associated with human FOXP3^+^-Tregs, although none of these molecules is exclusive for this subset, but shared with activated conventional T cells. To date, the most reliable feature unambiguously identifying FOXP3^+^-Tregs is the epigenetic remodeling of specific genomic regions within the *FOXP3*-locus (CNS2-TSDR) (24) or in Treg-related genes (11).

FOXP3^+^-Tregs modulate both innate and adaptive immune cells by various mechanisms. The inhibitory activity of Tregs is primarily dependent on contact with target cells, which allows modulation of antigen-presenting cells stimulatory capacity *via* CTLA-4 (25) or the killing of T effector (Teff) cells through the granzyme/perforin axis (26, 27). Additional mechanisms of suppression include the release of inhibitory cytokines, e.g., IL-10 (28), TGF-β (29, 30), and IL-35, at least in murine Tregs (31), cytokine deprivation (32), and generation of immunosuppressive metabolites, i.e., extracellular adenosine (33) and intracellular cAMP (34). FOXP3^+^-Tregs are not a homogeneous population but are rather constituted by a heterogeneous pool, including specialized subtypes (28, 35–39).

Their great potential as modulators of immune responses, resulting from both preclinical models and clinical evidences, convinced investigators that Tregs could be used as tools to control unwanted immune responses in the context of transplantation or to treat autoimmune/inflammatory diseases (40, 41). A great effort has been devoted to the development of good-manufacturing practice-grade protocols to isolate/expand human Tregs *in vitro* allowing translation of Treg-based cell therapy to the clinical practice (42–45).

In this review, we will give an overview of the clinical trials that applied FOXP3^+^-Tregs as therapeutics for the control of graft-versus-host disease (GvHD) in the context of hematopoietic stem cell transplantation (HSCT) and for the modulation of autoimmune reactions and the challenges that these trials highlighted. We will discuss the innovative therapeutic approach based on adoptive transfer of engineered Treg-like cells that we are developing for the treatment of IPEX syndrome, whose application could potentially extend to reestablish tolerance in autoimmune diseases of different origin and in transplantation.

## Treg-Based Cell Therapy in Clinical Trials

Several Phase I-clinical trials have been conducted to assess the effect of Treg-based cell therapy on GvHD following allogenic HSCT, organ transplantation, in patients with type 1 diabetes (T1D) and chronic inflammatory diseases. Overall, results obtained with different subsets of Tregs demonstrated favorable safety profiles (46, 47).

Regulatory T cell-based clinical trials in HSCT have preceded other indications because the timing of GvHD onset is known and can be monitored, the time needed for prevention is relatively short, the initial efficacy is likely to provide lifelong protection, and complications of GvHD can be lethal.

Several groups have applied polyclonal CD4^+^CD25^+^ Tregs containing a high proportion of FOXP3^+^ T cells, either freshly isolated or *ex vivo* expanded, with the aim of preventing GvHD after allogenic HSCT for onco-hematological diseases. The results showed that the overall procedure is feasible and safe (48–52). One trial reported decreased incidence of grade II–IV GvHD as compared with historical controls in patients receiving umbilical cord blood-derived Tregs, without increased infections (49). Data were confirmed in a more recent trial from the same group, in which the clinical outcome of patients receiving Treg-based cell therapy was compared with that of control patients who received the same conditioning regimen and immunosuppressive treatment but no Tregs. The incidence of grade II–IV acute-GvHD at 100 days was 9 vs 45% in controls, whereas chronic-GvHD at 1 year was 0 in treated patients (52).

In a third trial patients injected with freshly isolated peripheral Tregs showed low grade GvHD and no development of chronic-GvHD (50). More recently, the same group showed reduced incidence of relapse in Treg-treated patients (53).

These initial reassuring results encouraged a wider application of Tregs as therapy after solid organ transplantation. Several trials are currently ongoing, although final results are not currently available (47). Among those, in The-ONE-Study (http://www.onestudy.org/), a Phase I/II dose-escalation study, several subtypes of Tregs, including *ex vivo* expanded FOXP3^+^-Tregs, have been infused in patients undergoing kidney transplant with the goal of avoiding lifelong immunosuppression through the induction of active tolerance (NCT02129881) (47, 54). Similarly, a Treg-immunotherapy trial in the setting of liver transplantation, ThRIL (NCT02166177), has been initiated, although safety data are not yet available (44).

FOXP3^+^-Treg-based therapy was safely tested also in the context of autoimmune diseases. In a trial limited to few patients, *ex vivo* expanded CD4^+^CD25^hi^CD127^−^ Tregs were administered to children with recent-onset T1D (55), and more recently to new-onset adult T1D patients (43). In both cases, the procedure appeared to be safe, although published data do not allow to draw conclusions on efficacy. Importantly, in the latter trial, safety was demonstrated for transfer of high number of Tregs (up to 2.6 × 10^9^ cells) (43).

Overall, the data available support the feasibility and safety of the approach. These results convinced researchers to pursue adoptive Treg-cell therapy and much effort is currently devoted to address open issues in the field, such as the *in vivo* persistence and stability of the injected product and the need for Ag specificity to increase efficacy.

## IPEX Syndrome: A Disease Model of Treg Dysfunction

Immune-dysregulation, polyendocrinopathy, enteropathy, X-linked syndrome is the prototype poly-autoimmune disease caused by mutations in the gene encoding for the transcription factor FOXP3 (8). Affected patients develop early-onset multi-organ autoimmunity, which includes severe enteropathy, T1D, and eczema (9, 56). Beside the severely affected patients, many subjects manifest with a milder form of the disease, which is often misdiagnosed or diagnosed later due to the atypical presentation (57). *FOXP3* mutations result in loss of functional Tregs, which is considered the primary cause of disease. *FOXP3*-mutated Tregs display defective *in vitro* suppressive function (58–60) and unstable behavior in inflammatory conditions, with conversion from a regulatory to an effector (i.e., IL-17-producing) phenotype (61). Defects in peripheral cells other than Tregs have also been described, e.g., conventional T cells (58, 61–63) and B cells (64). Those additional defects are likely to be an indirect consequence of Treg dysfunction, rather than a direct effect of the mutations, thus suggesting that therapies aimed at improving/restoring a functional Treg compartment should be beneficial to IPEX patients.

The treatment of IPEX syndrome currently relies on supportive therapy, immunosuppression, and HSCT. Allogenic HSCT has proven curative (9), but for patients who do not undergo HSCT the treatment is limited to nutritional support, replacement therapy for endocrine organ failure, and to multiple immunosuppressive drugs, with incomplete control of autoimmunity and burdensome side effects in young patients. Therefore, a therapy aimed at restoring Treg functions represents an unmet medical need. Furthermore, experimental evidence in *scurfy* mice, the murine model of FOXP3-deficiency, shows that adoptive Treg transfer improves lifespan (65). On the same line, experience from transplanted patients with partial donor chimerism (66–68) and the presence of a fully wild-type Treg compartment in healthy carriers of *FOXP3* mutations (69) supports the idea that few functional Tregs are sufficient to control disease progression and induce remission.

The latter evidences convinced us that restoration of a functional Treg compartment in IPEX patients is a therapeutic option. We therefore designed an approach to genetically modify autologous T cells for adoptive transfer in these patients.

## The Generation of Treg-Like Cells by Lentivirus-Mediated FOXP3 Gene Transfer

The genetic reprogramming of mammalian cells for clinical purposes has recently become an available option, with the completion of clinical trials for the treatment of genetic diseases (70–73) and cancer (74, 75) and their translation in market-authorized therapies (76). Gene-transfer technology has been applied also to the field of Treg-based cell therapy, with the aim of generating high numbers of functional Tregs. Ectopic overexpression of FOXP3 in conventional CD4^+^ T cells from healthy donors (3, 77–79), ectopic expression of T cell receptors (TCRs) with known specificity in polyclonal Tregs (80–82), and the use of chimeric antigen receptors (CARs) (83–86) are the approaches so far tested in preclinical settings (Table 1). While the former approach would maintain the Ag specificity of the starting population, the latter would redirect Treg specificity. In preclinical studies, expression of TCRs specific for tumor-Ags/allergens conferred human Tregs the ability to suppress Ag-specific responses (80, 81). More recently, Tregs-expressing CARs specific for HLA-Ags have proven effective in inhibiting xenogeneic GvHD and allograft rejection in preclinical models (84–86).

**Table 1:** *In vitro* generation of genetically engineered human regulatory T cells (Tregs).

Cell type	Starting cell population	Gene-transfer platform/transgene	Marker gene	Ag specificity	*In vitro* suppression	*In vivo* function	Disease indication	Reference
CEA-CAR Tregs	Bead-sorted CD4^+^CD25^hi^ Tregs	RV/CEA-CAR	No	CEA	–	CEA + CD15A3 cell tumor model	–	(83)
A2-CAR Tregs	FACS-sorted naïve CD4^+^CD25^+^ Tregs	Bidirectional LV/A2-CAR	Yes (ΔNGFR)	HLA-A2	Yes	Xeno-GvHD model	Transplantation	(84)
MHC-I-allospecific-CAR Tregs	Bead-purified CD25^+^ Tregs	LV/A2-CAR	Yes (eGFP)	HLA-A2	Yes	Skin xenograft transplant	Transplantation	(85)
A2-CAR Tregs	FACS-sorted CD4^+^CD25^hi^CD127^low^ Tregs	RV/A2-CAR	Yes (ΔNGFR)		Yes	*In vivo* MLR; Hu-mice rejection model; Skin xenotransplant	Transplantation	(86)
Tyr-TCR Tregs	FACS-sorted CD4^+^CD25^hi^CD127^low^ naïve Tregs	Multicistronic LV/Tyr-specific TCR chains	Yes (GFP)	Tyrosinase	Yes	EL-4-HLA-A2/K tumor model	–	(80)
Islet Ag-specific Tregs	FACS-sorted CD4^+^CD25^hi^CD127^low^ Tregs	Multicistronic LV/Islet Ag TCR chains	Yes (mCherry)	IA2, insulin	Yes	–	T1D	(82)
Betv1-TCR-Tregs	Bead-sorted CD4^+^ CD25^−^ T cells	RV/Betv1-specific TCR chains and FOXP3	No	Betv1	Yes	–	Allergy	(81)
CD4^FOXP3^	Bead-sorted CD4^+^ T cells	Bidirectional LV/FOXP3	Yes (ΔNGFR)	Polyclonal	Yes	Xeno-GvHD model	IPEX syndrome	(79, 87)

*CAR, chimeric antigen receptor; A2, HLA-A2; GvHD, graft-versus-host disease; LV, lentiviral vector; RV, retroviral vector; CEA, carcinoembryonic antigen; MLR, mixed lymphocyte reaction; FOXP3, forkhead-box-P3; IPEX, immune-dysregulation, polyendocrinopathy, enteropathy, X-linked; T1D, type 1 diabetes; TCR, T cell receptor*.

**Figure 1:**
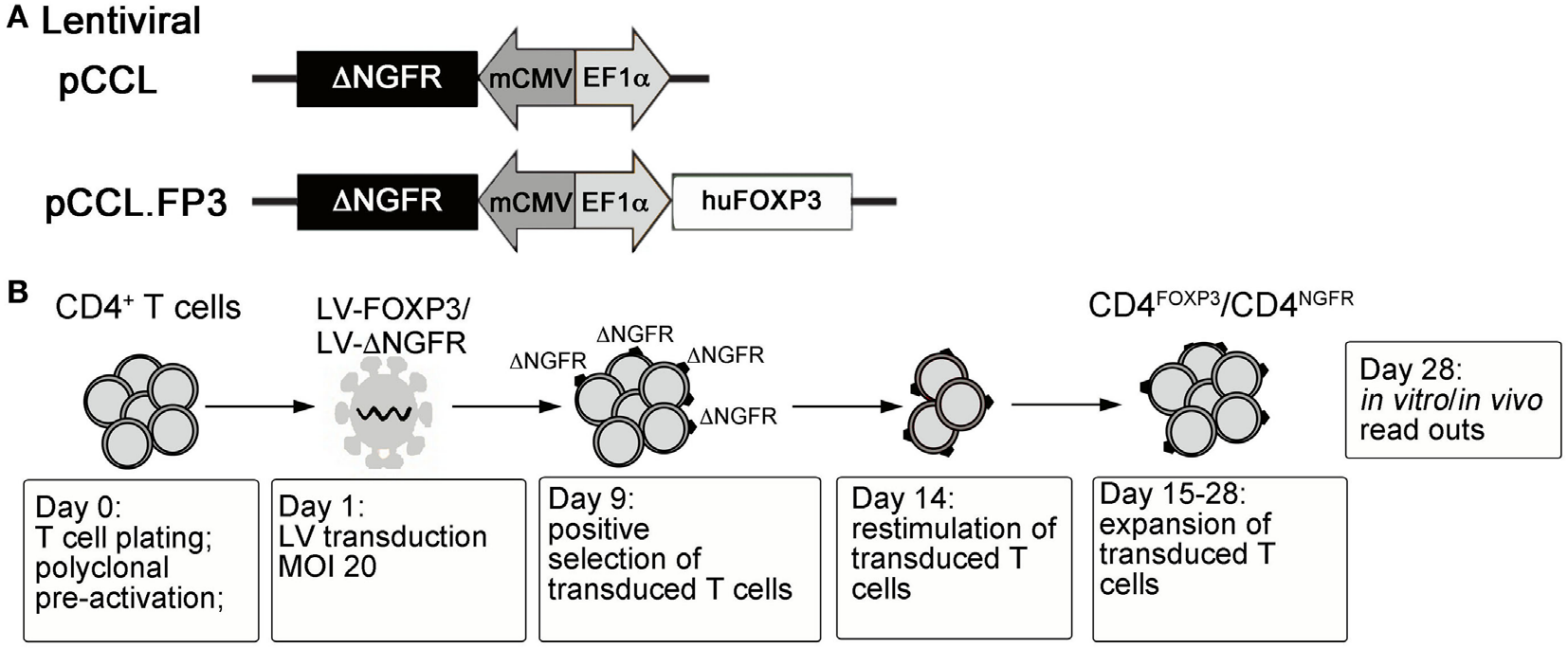
**(A)** Schematic representation of control and FOXP3-expressing lentiviral vector constructs. **(B)** Protocol for the generation of CD4^FOXP3^ Treg-like cells and control transduced T cells (CD4^NGFR^) from conventional CD4^+^ T cells (either naïve or total CD4^+^ T cells). MOI, multiplicity of infection; FOXP3, forkhead-box-P3; Treg, regulatory T cell.

With the ultimate goal of controlling the devastating autoimmunity resulting from mutations of *FOXP3* in IPEX syndrome, we envisaged the possibility of performing adoptive transfer of functional autologous Tregs generated *in vitro*. To this aim, the human *FOXP3* coding sequence was cloned under the control of a constitutive promoter in a bidirectional lentiviral vector (LV) construct (88) allowing simultaneous expression of full-length FOXP3 and of a cell-surface marker (ΔNGFR) for the identification/selection of transduced T cells (79) (LV-FOXP3) (Figure 1A). Transduction of peripheral CD4^+^ T lymphocytes with LV-FOXP3 and *in vitro* expansion of transduced cells lead to the generation of a homogeneous pool of T cells constitutively overexpressing FOXP3 (Figure 1B). The resulting CD4^FOXP3^ T cells behave as functional and stable FOXP3^+^-Treg-like cells, with potent *in vitro* suppressive activity, reduced proliferative capacity, and limited cytokine production (79, 87). CD4^FOXP3^ T cells stably express FOXP3 in steady-state and inflammatory conditions, especially when generated from naïve T cells, and maintain inhibitory functions *in vivo* in a model of xenogeneic GvHD (87). Furthermore, we demonstrated that fully functional CD4^FOXP3^ T cells can be generated from T cells of IPEX patients (87), regardless of the underlying *FOXP3* mutation and co-expression of mutated protein, thus demonstrating the feasibility of our approach and paving the way for the development of alternative therapies based on the adoptive transfer of autologous genetically modified Treg-like cells for the control of autoimmunity in IPEX syndrome.

The fact that CD4^FOXP3^ T cells can be obtained from CD4^+^ T cells renders the manufacturing process easy and cost-effective. CD4^FOXP3^ T cells do not require extensive *in vitro* expansion with high cytokine concentration. The current preclinical small-scale method for the generation of CD4^FOXP3^ T cells leads up to 10-fold expansion in 3/4-week culture. This guarantees the feasibility of the production for infusion into patients, taking into consideration that the starting conventional CD4^+^ T cells can be available in large numbers. In addition, the clinical use of LV platforms does not pose a limitation, since it has proven to be safe in cancer patients and pediatric patients who received HSC gene therapy (72, 73, 89, 90).

Although in principle, the use of CAR-Tregs or TCR-transgenic Tregs would allow the generation of Ag-specific Tregs suitable for the treatment of autoimmune diseases, the Ag target of the autoimmune damage is still unknown in many diseases. The fact that CD4^FOXP3^ originate from polyclonal CD4^+^ T cells may constitute an advantage for such diseases. Indeed, the CD4^+^ T cells obtained from a patient suffering with autoimmunity would most likely comprise the pathogenic T cells with TCRs specific for the target Ags. Therefore, in specific disease context, CD4^FOXP3^ cells may find a broader and more effective use, as compared with the TCR-transgenic-/CAR-Tregs.

## Challenges in Treg-Based Immunotherapy for IPEX Syndrome

Despite their promising results, the initial trials of Treg-based cell therapy raised some concern on issues related to FOXP3^+^-Treg biology. Due to their intrinsic anergic and terminally differentiated phenotype, one open issue is the *in vivo* lifespan of the infused product. Initial data on *in vivo* infused Tregs showed 2-week survival post-injection (49). We obtained similar results when CD4^FOXP3^ T cells were injected in immune-deficient mice (87). Surprisingly, data from a Treg-cell therapy trial in T1D patients demonstrated that, although the majority of *ex vivo* expanded autologous Tregs persists for 2 weeks post-infusion, a fraction of the injected cells is detectable after 1 year, suggesting that Tregs might contribute to tolerance maintenance long term (43).

Several evidences demonstrated that FOXP3^+^-Tregs are intrinsically plastic and that under inflammatory conditions Tregs can downmodulate FOXP3 and secrete pro-inflammatory cytokines (91–93). Therefore, the risk of loss of regulatory functions by infused Tregs could be worrisome. To address this issue, culture with rapamycin, to favor the generation of stable Treg products, has been developed (94–96). In this context, CD4^FOXP3^ T cells represent the ideal Treg product: constant FOXP3 expression is warranted by a constitutive promoter-driven transcription, and stability has been demonstrated in steady-state and inflammatory conditions, both *in vitro* and *in vivo* (87). Stability is especially maintained when CD4^FOXP3^ T cells are generated from naïve T cells. In the case of memory-derived CD4^FOXP3^ T cells, FOXP3 expression appeared slightly reduced with inflammatory cytokines, resulting in weaker suppressive function and increased proliferation, as compared with naïve T cell-derived products (87), most likely due to posttranscriptional regulatory mechanisms.

Finally, the possibility of a generalized effect of immunosuppression that injection of suppressor cells may cause, as well as concerns about the dose required for injection of polyclonal Tregs has prompted investigators to design more targeted therapies. Methods to expand human Ag-specific Tregs have been developed (42, 97, 98). These protocols well apply to allo-Ag-specific Tregs. Of note, encouraging safety and efficacy results come from a recently published Treg-based cell therapy trial, in which Tregs induced in the presence of donor-irradiated PBMCs were infused after liver transplantation. Despite low doses of Tregs, in 7/10 patients, immunosuppression was stopped, and operational tolerance to the graft was induced (99). Currently, ongoing trials in solid organ transplantation, which foresee the injection of donor-specific Tregs, will lead to further progresses (47).

We believe that in the case of IPEX syndrome and diseases with multiple autoimmune manifestations, the need for Ag specificity is unlikely to be necessary. The use of patients’ Teff cells as source of CD4^FOXP3^ cells will potentially allow the generation of Treg-like cells enriched for autoreactive specificities. Still, the infusion of polyclonal potent suppressor cells may result in a generalized effect of immunosuppression, which could potentially interfere with protective responses to common pathogens. Although the results of the clinical trials using polyclonal Tregs were reassuring, we are currently establishing a protocol to generate CD4^FOXP3^ T cells from Ag-experienced T cells with known specificity, which should restrict their suppressive effect to the target Ag. Briefly, the protocol foresees pre-activation of T cells with a target Ag; Ag-specific T cells activating in response to their cognate Ag will be preferentially transduced. Subsequent *in vitro* expansion allows generation of a T-cell population enriched of FOXP3-overexpressing cells with known Ag specificity (Passerini and Bacchetta, unpublished results). This method could be used to extend the application of the CD4^FOXP3^ T-cell product beyond IPEX syndrome, to treat autoimmune/inflammatory diseases with known target Ags or in the context of transplantation tolerance.

Finally, a relevant open issue on the way to the clinical application of CD4^FOXP3^ T cells is definitely their *in vivo* lifespan, difficult to assess in preclinical models. Short-lived cells would likely be safer, although they may imply clinical protocols with multiple infusions of the therapeutic product. Long-lived CD4^FOXP3^ T cells would allow single infusion but would likely require an additional safety layer, such as addition of a suicide gene in the construct used for their generation. The use of a suicide gene may also be considered as a safety measure to contrast the consequences of possible insertional mutagenesis, although it has been demonstrated that the use of LV-mediated gene transfer is not associated with selective integrations near oncogenes (100). However, for any type of genetically modified cellular product, analysis of the integration sites is recommended during preclinical assessments.

## Concluding Remarks

Thanks to the successfully completed trials, the use of adoptive Treg-cell therapy to control undesired immune responses has become applicable. The next challenge for researchers is the tailoring of the Treg-based therapy for specific diseases. We envisaged an approach based on the use of FOX3^+^-Treg-like cells electively designed to restore immune regulation in IPEX syndrome. Once safety and proof-of-concept will be completed in IPEX patients, the use of these autologous Treg-like cells could become the future standard of care for certain autoimmune diseases, akin to how CAR-T cells will become the standard of care in hematologic malignancies.

## Author Contributions

The authors (LP and RB) equally contributed to discuss the topic and write the manuscript.

## Conflict of Interest Statement

The authors declare that the research was conducted in the absence of any commercial or financial relationships that could be construed as a potential conflict of interest.
